# Preventing catheter-related infections in ICUs: comparing catheter care techniques

**DOI:** 10.1186/cc14152

**Published:** 2015-03-16

**Authors:** S Ozden, R Iscimen, H Akalin, N Kelebek Girgin, F Kahveci, M Sinirtas

**Affiliations:** 1 Uludag University Faculty of Medicine, Bursa, Turkey

## Introduction

Catheter-related bloodstream infections (CRBSI) are common and an important cause of morbidity and mortality in critical patients. Optimum approaches for preventing infections are presented in guidelines. This study aims to evaluate efficacy of different care techniques and education, to define risk factors for decreasing ratio of CRBSIs and to analyze effects on morbidity and mortality.

## Methods

After ethics committee approval, patients admitted to the ICU, older than 18 years, who were thought to have a central venous catheter (CVC) for more than 48 hours, and whose first catheter was inserted in the ICU were included in the study. Staff were educated before the study and periodically during the study. Catheter care and insertion were applied according to the guidelines. The study was planned as three sequences. In the first group, catheter care was made with a sterile gauze pad. In the second and third groups, catheter care was made with chlorhexidine gluconate impregnated dressing. Also in the third group, a silver-coated needleless connector was inserted into the tip of venous catheters.

## Results

Totally 105 patients were included in the study and every group included 35 patients. There was no difference between groups when evaluating reasons for catheter insertion. There was no statistically significant difference according to emergent or elective catheterization, trying times, or catheter insertion side (*P *> 0.05). CRBSI was determined in two patients in group 1, in one patient in group 2, and in no patient in group 3. In group 1 it was observed on the 4th and 11th days. In group 2 it was observed on the 18th day after catheterization. Before the study, a statistically significant decrease was determined in CRBSI ratios before and after education (16.4/1,000, 12.9/1,000 catheter-days (*P *< 0.001)). According to Group 1 a statistically meaningful decrease was assigned in CRBSI ratios in Groups 2 and 3 (4.84/1,000, 2.22/1,000, 0/1,000 catheter-days) (*P *< 0.001, *P *< 0.001, *P *< 0.001).

## Conclusion

Continued education is important in preventing CRBSIs. Maximum precautions must be taken. Usage of antiseptic solutions with clorhexidine and chlorhexidine gluconate impregnated dressing decreased insertion side infections and usage of silver-coated needleless connectors reduced microorganism entry through the catheter lumen and provided a severe decrease in infection ratio.
